# Validation of a Portuguese version of the Groningen radiotherapy-induced xerostomia questionnaire

**DOI:** 10.4317/medoral.25428

**Published:** 2022-09-29

**Authors:** Filipe Freitas, David Braz, Ruben Pereira, Daniel de Sousa, Duarte Marques, João Caramês, António Mata

**Affiliations:** 1 DDS, PhD. Faculdade de Medicina Dentária, Universidade de Lisboa, Lisbon, Portugal; 2 DDS. Faculdade de Medicina Dentária, Universidade de Lisboa, Lisbon, Portugal; 3 MD, PhD. Instituto Português de Oncologia de Lisboa, Lisbon, Portugal; 4 Oral Biology and Biochemistry Research Group, Biomedical and Oral Sciences Research Unit, Lisbon, Portugal; 5 LIBPhys – FCT UID/FIS/04559/2013, Lisbon, Portugal

## Abstract

**Background:**

The aim of this study was to validate and determine at pretest level the reliability of the Portuguese version of the Groningen radiotherapy-induced xerostomia questionnaire.

**Material and Methods:**

This study employed 37 head and neck cancer patients. Each patient signed an informed consent and responded to the Portuguese version of the questionnaire in the form of an interview. This was repeated again after 2 weeks. A standard single question provided a validity check. Data were analyzed using Cronbach’s α to test its reliability and total and interitem correlation, and intraclass correlation to determine its internal consistency and test-retest reliability. Construct validity supported by objective measurements as salivary secretion was also investigated. Significance was set at .05.

**Results:**

Cronbach’s α was 0.91 and 0.89 for the first and second test administrations, respectively, which indicates that the internal consistency was excellent. The intraclass correlation coefficient value for the test-retest reliability was 0.70. The correlation between the total score of the questionnaire and standard single dry mouth question was 0.72 for the first round, indicating a good correlation.

**Conclusions:**

Demonstrating very good psychometric properties, the Portuguese version of the Groningen radiotherapy-induced xerostomia questionnaire is a valid tool and can be considered a reliable instrument to measure xerostomia in head and neck cancer patients.

** Key words:**Xerostomia, quality of life, xerostomia questionnaire, transcultural validation, head and neck cancer, radiotherapy, saliva.

## Introduction

Head and neck cancer (HNC) is the sixth most common cancer worldwide and is often managed with radiotherapy, either as monotherapy or in association with chemotherapy and surgery (1). Irradiation of the salivary glands may result in salivary hypofunction (i.e., diminished salivary flow) and subsequent xerostomia (i.e., the subjective sensation of a dry mouth), which is one of the most frequently reported side effects of radiation treatment in the head and neck area (2-4). It has been calculated that 93% of patients experience xerostomia during head and neck radiotherapy, and that 74% to 85% of patients experience xerostomia one month to two years postradiotherapy, respectively (5). The profound salivary gland dysfunction and xerostomia often observed in response to external radiotherapy in the head and neck region may have a massive impact on patient’s oral health and oral health-related quality of life (QoL) (5,6). From this point of view, xerostomia as reported by patients may provide important additional information in the assessment of radiation-induced salivary gland dysfunction. Therefore, it is important to use a validated xerostomia assessment scale, and a validated questionnaire specifically addressing the impact of xerostomia on QoL aspects (7-9).

The EORTC QLQ-C30 and the EORTC QLQ-H&N35 are the most commonly used validated questionnaires to determine HRQOL after irradiation of head and neck cancer in clinical trials (10-12). The EORTC QLQ-H&N35 contains 35 questions concerning treatment-related symptoms and symptoms frequently present in head and neck cancer patients. As this questionnaire only contains one item about xerostomia and one item about sticky saliva, the question arises as to whether it is sufficiently sensitive to score more discrete changes of patient-rated xerostomia. In addition, the QLQ-H&N35 does not allow for the assessment of different aspects of xerostomia at different time points (13). Some patients mainly suffer from xerostomia at night while others have complaints predominantly during the day (8). Content and production of saliva may differ among different salivary glands and show a circadian rhythm, which may have various impacts on different aspects of symptoms related to salivary dysfunction (14,15). Therefore, it was developed the Groningen radiotherapy-induced questionnaire (GRIXQ), a new questionnaire that enables scoring of different aspects of patient-rated xerostomia. It can also be used to evaluate the impact of emerging radiation delivery techniques aiming at prevention of xerostomia in more detail (13).

## Material and Methods

The aim of this study was to develop a Portuguese version of the Groningen radiotherapy-induced xerostomia questionnaire (GRIXQ-PV) and assess its psychometric characteristics.

- Transcultural adaptation

The original GRIXQ is composed of 14 questions from which the respondent can choose from 4 available answers: “not at all” (scoring 1), “a little” (scoring 2), or “quite a bit” (scoring 3) or “very much” (scoring 4). The scores from the 14 items are summed up to originate a final value that can range from 14 to 56. The result representing the degree of xerostomia the subject feels, with higher scores imply greater severity in symptoms.

The questionnaire was adapted following the guidelines for cross-cultural adaptation on health-related measures comparing semantic, idiomatic, experiential, and conceptual equivalence (16-18). The resulting Portuguese version was read and commented upon by three different dentists from the filed of oral medicine. The revised version of the GRIXQ-PV is depicted in Table 1.

- Patients and the intervention

This study employed a convenient and consecutive sample of 37 patients with head and neck cancer who were previously recruited for a randomized clinical trial on gustatory stimulants of salivary secretion at the Portuguese Institute of Oncology in Lisbon. The inclusion criteria for this study were as follows: (i) head and neck cancer patients treated with radiotherapy; and (ii) above 18 years of age. Exclusion criteria were as follows: (i) wearer of complete dental prosthesis; (ii) those who were pregnant or lactating; and (iii) non- speakers of Portuguese.

Written informed consent was obtained from all eligible participants as the first sage of screening and before study admission. A full medical history was taken, and saliva collection was performed expressly for this study to determine and evaluate construct validity, by established methods (19,20).

Each patient answered to the GRIXQ-PV version of the questionnaire in the form of a standardized interview. Study participants were asked to indicate which 1 to 4 response options best described their symptoms over the preceding 2 weeks. They were instructed to give the answer that immediately came to mind and to request the interviewer for additional clarification or to repeat the question if they could not understand before providing a response. This procedure was repeated with a 2-week interval, to evaluate the test-retest reliability of the GRIXQ-PV.

Participants were also asked to respond “never,” “occasionally,” “frequently” or “always” to the single item: “How often does your mouth feel dry.” This was done to provide a concurrent validity check.

The ethical committees of the participating institutions approved the study protocol, which was conducted in full compliance with the World Medical Association Declaration of Helsinki and its most recent amendments and always followed good clinical practice guidelines.

**Table 1:**
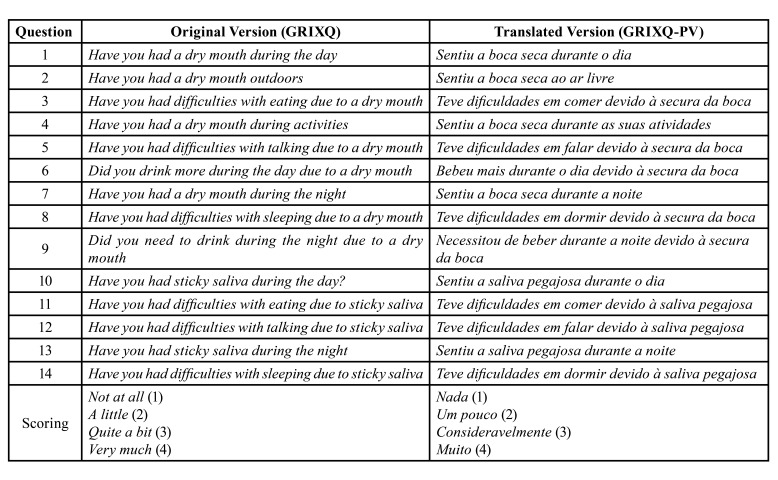
Original and Portuguese versions of Groningen radiotherapy-induced xerostomia questionnaire.

- Statistical analyses

A preestablished plan recurring to a statistical package (version 22.0; SPSS Inc., Chicago, IL, USA) was employed to analyze all data. Missing values were substituted by item question mean obtained from all the other questionnaires. If a patient failed to answer more than two questions was removed from the study. The dependent variable was the GRIXQ-PV score, expressed as the summated score ± SD. Significance was set at α = 0.05.

Internal consistency of the GRIXQ-PV was assessed by calculating Cronbach’s alpha. As defined previously for clinical studies, values at least 0.80 were considered desirable and rated as good (21). Despite the questionnaire is not very lengthy with 14 questions, inter-item correlations were calculated to determine the possibility of inflation of the Cronbach’s α value (22). For the scales to be considered sufficiently reliable for use in groups of patients, this value should be above 0.4, although 0.2 could be accepTable (23,24). We also examined correlations of all items with the overall score (item-total correlation), which should be above 0.3 and also if by removing a question, the value of Cronbach’s alpha would be improved (24).

After the determined 2-week interval, each patient was administrated once again the GRIXQ-PV. The procedure was identical to the first round. The test-retest reliability of the total score and subscore for every question was assessed by calculating intra-class correlation coefficient (ICC). The model used was two-way random with absolute agreement and 95% confidence intervals. ICC lower than 0.4 was considered to have poor reliability while a range from 0.4 to 0.75 has fair to good reliability. The optimal ICC values should be higher than 0.75 to an excellent reliability.

To determine the construct validity of the Portuguese version of the questionnaire, aspects of the convergent validity were considered. Thus, relationships were examined between GRIXQ-PV scores and other measures that are assumed to be derived from the same construct. Therefore, total GRIXQ-PV scores were plotted in function of resting, stimulated, and differential (stimulated minus resting) salivary flows, and Pearson correlations analysis was obtained. It was hypothesized a priori that a negative correlation existed between saliva production and xerostomia reporting. Pearson’s coefficient was interpreted as follows: strong correlation for values > 0.50; moderate correlation for values between 0.35 and 0.50; weak correlation for values < 0.35.

The means of the total GRIXQ-PV scores were also plotted against the standard question response categories to assert concurrent validity. The correlation between the total scores and the standard question responses was examined using Spearman’s ρ.

Floor and ceiling effects were a concern for the assessment of content validity. These should deem to be influencing the questionnaire if more than 15% of the participants scored in the extremes of the overall summated score (25).

## Results

Whereas translation procedures were concerned no difficulties were encountered. Idiomatic equivalences were discussed, and consensus reached swiftly between members of the translating panel. The final version was unanimously found to be perfectly understood by any Portuguese speaking person.

No patients had to be discarded from the study for missing more than two questions. One patient had one question missed in which the value was replaced with the average values of the other answers from the test, as previously described.

Data on the demographic and salivary characteristics of the data set are presented in Table 2. Age and gender characteristics are accordingly with the previously described for head and neck cancer patients (1).

The mean scores of the 14 questions of the test as its total score are shown in Table 3. Mean total GRIXQ-PV scores and standard deviation (s.d.) were 26.78 ± 9.496 and 27.97 ± 9.317 for first test administration and 2-week delayed repetition, respectively. Total GRIXQ-PV scores ranged from 14 to 47. No patients scored the maximum score of 55 and only 3 patients on the first visit and 2 on the second scored the minimum value of 14. Therefore, floor or ceiling effect was not found.

Data on internal consistency and test-retest reliability are presented in Table 4. Cronbach alpha values for the 14 questions were 0.91 and 0.89 for both administrations, respectively. Inter-item correlation coefficient was of 0.42 and 0.37 in each visit. The item-total correlations and contribution for scale stability and variance are also presented. The results showed a similar and homogeneous contribution for scale dimensionality for each item in the scale. Scores for both questionnaire administration and ICC results showed good reliability with ICC for the total score of 0.67.

**Figure 1:**
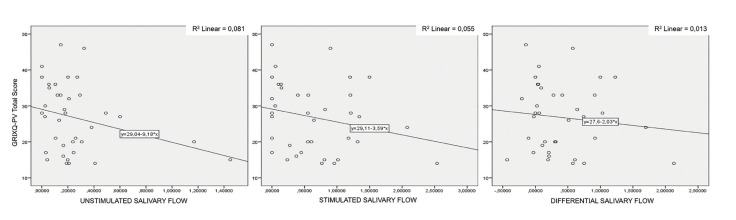
Scatter plots of total GRIXQ-PV scores of the first round in function of unstimulated, stimulated, and differential flows. Regression line and 95% confidence interval interpolation are displayed.

Pearson correlation coefficients between total GRIXQ-PV score and resting, stimulated, and differential salivary flows were -0.284, -0.234, and -0.115, respectively (with the 0.089, 0.163, and 0.498 significance levels). The results showed a negative, but poor and not significant correlation between total GRIXQ-PV score and salivary flows. Scatter plots of total GRIXQ-PV scores in function of resting, stimulated, and differential salivary flows are depicted in Fig. 1.

**Figure 2:**
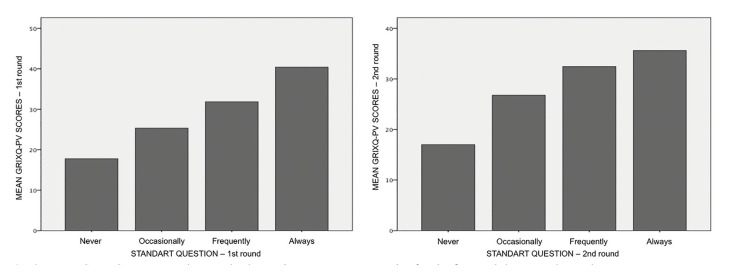
Mean GRIXQ-PV scores by standard question response categories for the first and the second round.

Finally, there was a strong positive correlation (Spearman’s ρ = 0.72 / 0.58) between the standard item response and the GRIXQ-PV total score for both rounds. Moreover, when plotting mean GRIXQ-PV scores by standard question responses, a statistically significant gradient across the categories of the standard question was observed, as seen in Fig. 2.

**Table 2:**
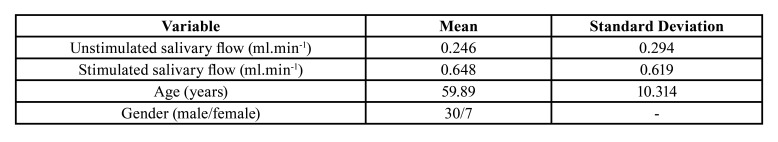
Demographic and salivary characteristics of sampled population (n=37).

**Table 3:**
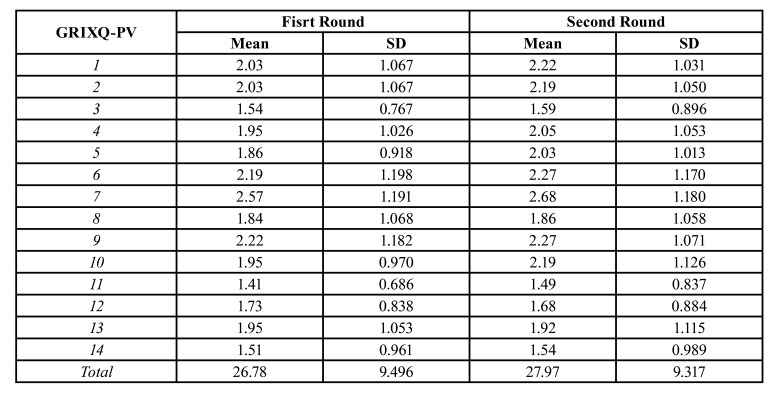
Mean scores and standard deviations of both administrations of the Portuguese version of the Groningen radiotherapy-induced xerostomia questionnaire.

**Table 4:**
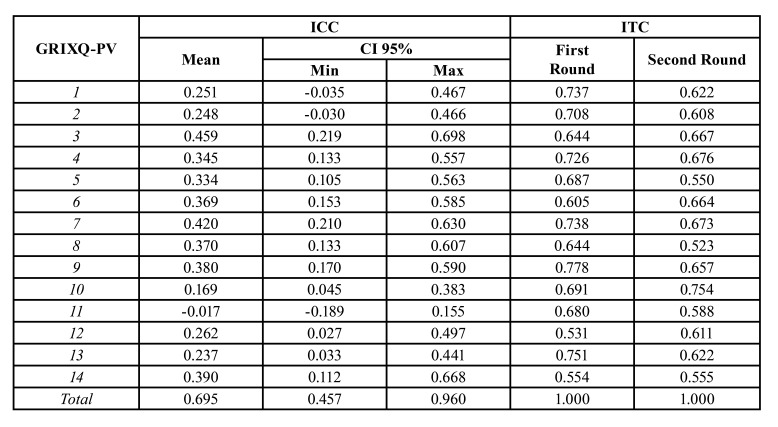
Intraclass correlation coefficient (ICC) and item-total correlation coefficient (ITC) for both administrations of the GRIXQ-PV.

## Discussion

This study was designed as a descriptive cross-sectional survey aiming at the translation of the GRIXQ into Portuguese and describes preliminary psychometric testing. A Portuguese version of the GRIXQ was obtained from the original version by established guidelines and administered twice at a 2-week interval to head and neck cancer patients. The main finding of this study is the suggestion that after pretesting, the Portuguese version of the GRIXQ seems to be a reliable and valid form of measure xerostomia similarly as its parent English version.

We are perfectly aware of the limitations of this study, mainly the size of the sample. However, this was a pretest of the GRIXQ-PV. Pretesting is an essential step in multicultural and linguistic adaptation of any version, with the objective of evaluating the translated version in a quick manner and then rediscussing it within the expert panel (17).

For testing construct validity, we investigated the total GRIX-PV score correlation with resting, stimulated, and a derived variable obtained from the difference between the former, which expressed the secretion capacity. We chose to do so because xerostomia most frequently arises from the diminishment of salivation and, therefore, could correlate with the patient’s own perception of this condition. This is controversial and may be viewed as a study weakness because some authors have reported low correlations between salivation and xerostomia (26,27). In our study, there was a negative but poor and not significant correlation between the total GRIXQ-PV score and the salivary flows.

Some studies use a single question consisting in a one-dimensional test for patient self-reported xerostomia and use it for validation check purposes (26,27). In this study, we found a significant positive and strong correlation between the single-item question and total score, fulfilling the criteria for independent validation as proposed in previous studies (28).

A major strength of this study was the double administration of the questionnaire with a separate time interval, thus enabling the first assessment of the test-retest reliability of the GRIXQ-PV. Intraclass correlation coefficient for the total score was 0.70 with a two-week interval indicating good time stability for the GRIXQ-PV. This is an important finding as the test-retest reliability of any questionnaire is a critical characteristic. Intra-class correlation coefficients are positives for all items, except for the item 11.

Cronbach alpha value for the 14 questions was 0.91/0.89 for both test administrations. In health-related studies, a Cronbach alpha coefficient over 0.8 is recommended for general internal consistency assessment, thus the score obtained in this study suggests a good internal consistency for the GRIXQ-PV and that the 14 questions are measuring the same construct. Similar findings have been obtained in original questionnaire (13).

Moreover, positive correlations between all items were found. The mean inter-item correlation was 0.42/0.37 for both rounds, respectively. According to the literature, a mean inter-item correlation of 0.15–0.20 is desirable for scales that measure broad characteristics, while values of 0.40–0.50 are required for scales tapping narrower ones (23). Some authors suggest that values above 0.20 could be considered accepTable (24).

Strong correlations (0.53-0.78 / 0.52-0.75) were also found when comparing an item and the rest of the scale (item-total correlation), all well above the recommended threshold (0.3) for including an item in a scale (24). All items correlated well with total score and were kept in the questionnaire contributing to its internal consistency.

## Conclusions

The existence of a Portuguese version of this questionnaire is important and new, because Portuguese is the fifth language in the world spoken by more than 240 million people, which confers the GRIXQ-PV a wide clinical and research application. Future studies should try to confirm validation of the GRIXQ-PV. In summary and within the limitations of this study, GRIXQ-PV seems to be a valid and reliable instrument for measuring specific xerostomia rating of irradiated patients complaints.
